# Multimodal imaging and photothermal/chemodynamic therapy of cervical cancer using GSH-responsive MoS_2_@MnO_2_ theranostic nanoparticles

**DOI:** 10.1186/s11671-023-03902-9

**Published:** 2023-09-29

**Authors:** Runrun Shao, Xiaofang Qiao, Linlin Cao, Jianliang Man, Lingyun Guo, Lanlan Li, Wen Liu, Lihong Li, Bin Wang, Lixia Guo, Sufang Ma, Boye Zhang, Haojiang Wang, Lili Yan

**Affiliations:** 1 College of Basic Medicine University, Shanxi Medical Univerity, Taiyuan, 030000 People’s Republic of China; 2 Henan Center for Drug Evaluation and Inspection, Henan, 450000 People’s Republic of China

**Keywords:** GSH responsive, Tumor microenvironment, MR/CT imaging, Chemodynamic therapy

## Abstract

The development of nanoparticles capable of inducing reactive oxygen species (ROS) formation has become an important strategy for cancer therapy. Simultaneously, the preparation of multifunctional nanoparticles that respond to the tumor microenvironment is crucial for the diagnosis and treatment of tumors. In this study, we designed a Molybdenum disulfide (MoS_2_) core coated with Manganese dioxide (MnO_2_), which possessed a good photothermal effect and could produce Fenton-like Mn^2+^ in response to highly expressed glutathione (GSH) in the tumor microenvironment, thereby generating a chemodynamic therapy (CDT). The nanoparticles were further modified with Methoxypoly(Ethylene Glycol) 2000 (mPEG-NH_2_) to improve their biocompatibility, resulting in the formation of MoS_2_@MnO_2_-PEG. These nanoparticles were shown to possess significant Magnetic Resonance Imaging (MRI) and Computed Tomography (CT) imaging capabilities, making them useful in tumor diagnosis. In vitro and in vivo experiments demonstrated the antitumor ability of MoS_2_@MnO_2_-PEG, with a significant killing effect on tumor cells under combined treatment. These nanoparticles hold great potential for CDT/photothermal therapy (PTT) combined antitumor therapy and could be further explored in biomedical research.

**Supplementary Information:**

The online version contains supplementary material available at 10.1186/s11671-023-03902-9.

## Introduction

Cervical cancer is the second most common malignant tumor in women worldwide and has a high mortality rate, especially when it is associated with human papillomavirus (HPV) [1]. Chemotherapy, radiotherapy, surgery and immunotherapy are the methods used to treat patients with cervical cancer. However, with the help of nanomedicine and novel delivery systems, more of the efficacy of these methods can be achieved. In this paper, we aim to investigate the capacity of nanomedicine in establishing new cervical cancer treatments. As a barrier for tumor cells, the tumor micro-environment [2] provides conditions for tumor cells to continue to multiply and evade the immune mechanism [3], which is also the main reason for the poor prognosis of tumor treatment. Hypoxia, low pH, endogenous H_2_O_2_, and GSH excess are essential features of TME [4]. Here, in response to the physicochemical properties of TME, the design of TME-specific activated nanoparticles is of great significance for imaging diagnosis and precise treatment of tumors. MnO_2_ is a promising inorganic nanomaterial with the advantages of simple preparation, low price, and environmental friendliness [5]. In addition, MnO_2_ can specifically respond to excess GSH in the TME. Glutathione (GSH), which is overexpressed in tumor cells, has strong scavenging effect on free radicals. The high-priced manganese is reduced to Mn^2+^ by GSH [6], and the generated Mn^2+^ reacts with excess H_2_O_2_ in the TME to produce cytotoxic ROS [7]. In the meantime, GSH is converted to oxidized glutathione (GSSH), reducing the clearance of reactive oxygen species. Mn element has MRI imaging function, so it has an excellent biological application prospect. However, MnO_2_’s therapeutic effect is often limited by low reactivity and insufficient endogenous H_2_O_2_ in tumors. To overcome these limitations, temperature can be used to accelerate the Fenton [8] or Fenton-like [9] reaction rate, making the design of nanoparticles with both PTT and CDT effects promising.

The Mo element in MoS_2_ has a high atomic number and can provide CT imaging [10], while MoS_2_ has good photothermal [11] properties and can provide PTT [12] and Photothermal imaging (PTI) properties, which has good biological application prospects. The ROS produced by CDT can also inhibit the overexpression of heat shock proteins, thereby improving the PTT effect [13].

Therefore, we designed MoS_2_@MnO_2_-PEG nanoparticles that possess both PTT [14] and CDT effects and the two treatment modalities promote each other to achieve better therapeutic results [15, 16].

**Scheme 1:**
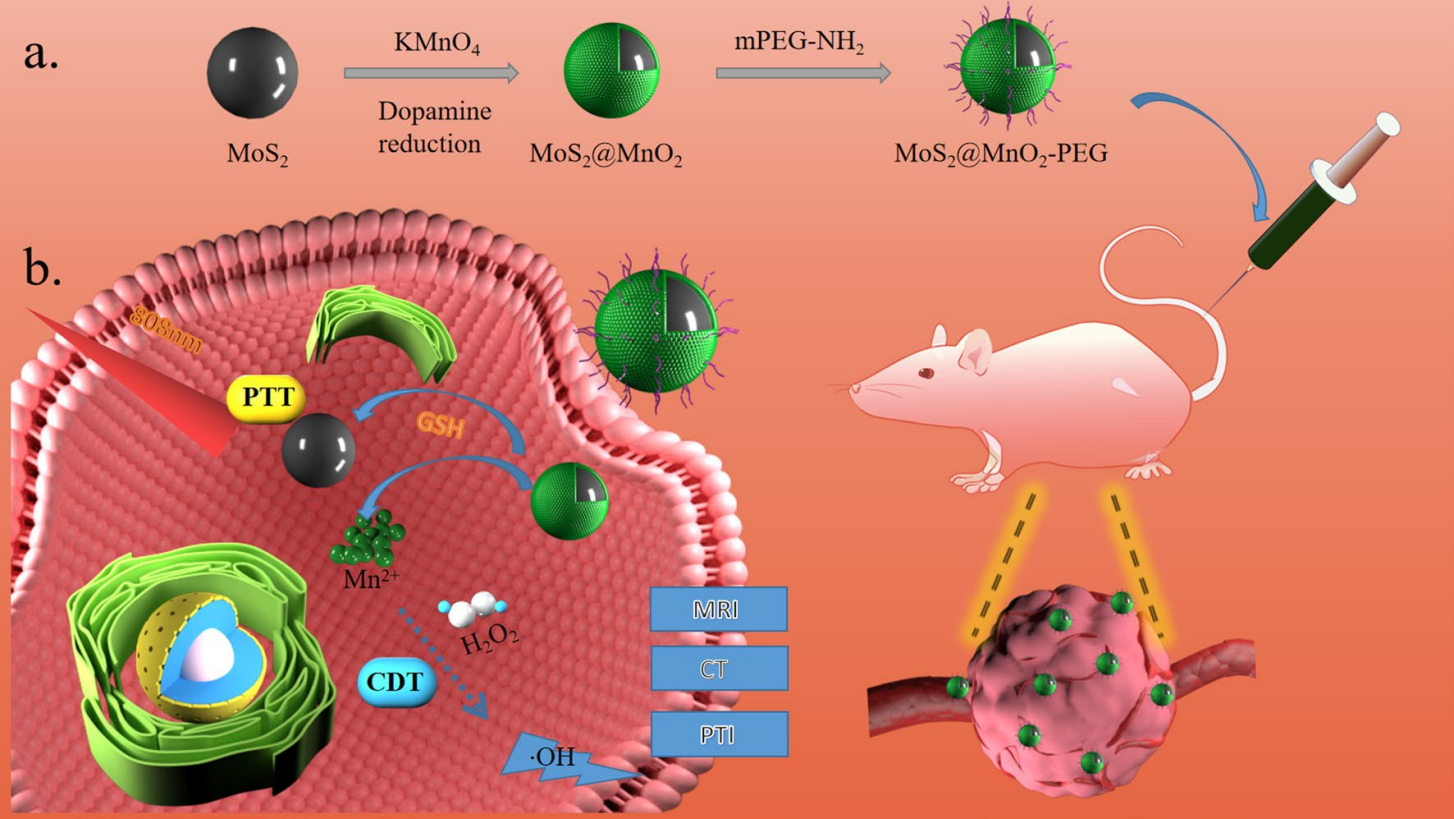
Schematic diagram of the preparation of MoS_2_@MnO_2_-PEG and the antitumor effect of CDT combined with PTT. **a** Preparation of MoS_2_@MnO_2_ and further polyethylene glycosylation reactions. **b** After intravenous injection of tumor-bearing mice, in vivo PTT combined with CDT treatment, including GSH depletion, Fenton-like response, and enhanced ROS production

TME possesses higher GSH [17] content than normal tissues, making it an attractive target for cancer therapy. In this study, we developed nanoparticles and a therapeutic system, MoS_2_@MnO_2_-PEG, that specifically respond to GSH in the TME [18]. The nanoparticles were designed with MoS_2_ as the skeleton structure, and MnO_2_ was wrapped on the surface using a simple hydrothermal reaction to form a mesoporous core–shell structure [19–21]. Next, to improve the stability and monodispersity of MoS_2_@MnO_2_ NSs in aqueous solution and facilitate their further biomedical utilization, mPEG-NH_2_ was used for the surface modification of MoS_2_@MnO_2_ NSs.The two react by electrostatic bonding. This structure possesses both PTT and CDT modes, and the two modes promote each other to achieve better therapeutic results [22–24]. Additionally, the nanoparticles provide CT, PTI, and MRI three-modal imaging [25, 26] functions, enabling accurate and effective tumor diagnosis and treatment [27]. Compared with previous materials, the nanoparticles synthesized in this study combine two imaging modes to realize multimodal imaging of nanomaterial [28].To improve the solubility and suspension stability of the nanoparticles in water, they were modified with polyethylene glycol (mPEG-NH_2_) to form MoS_2_@MnO_2_-PEG (Scheme 1). The modified nanoparticles showed significant antitumor ability in vitro and in vivo, making them a promising candidate for further biomedical research.

## Experiments

### Chemicals

Ethanol and ammonium hydroxide were purchased from DAMAO chemical reagent plants. Ammonium molybdate was provided by Sigma. Hydrazine hydrate, ethylene glycol, DA-HCl, KMnO_4,_ Rhodamine B (RhB), NaN_3_, and Methylene blue (MB) were purchased from Shanghai Aladdin Biochemical Technology Co Ltd. 3-(4,5)-dimethylthiahiazo(-z-y1)-3,5-di-phenytetrazoliumromide (MTT), Calcein-AM, PI and 2′,7′-dichlorofuorescin diacetate (DCFH-DA) were provided by Solarbio. No further purification was performed on any of the reagents.

### Preparation of MoS_2_@MnO_2_-PEG

Preparation of MoS_2_ involved dispersing 27.5 mg of ammonium molybdate in 12.5 mL of ethylene glycol solution through ultrasonic treatment for 10 min. Next, 147 μL of hydrazine hydrate (80%) was added, and the solution was subjected to ultrasonic treatment for 40 min and transferred to a 25 mL reactor for 10 h at 140 °C. After the reaction, the material was cooled to room temperature, washed with acetone three times, centrifuged for 5 min at 8000 rpm, and the pellets were collected and dried in a vacuum oven at 80 °C for 24 h.

To prepare MoS_2_@MnO_2_, 10 mg of dried MoS_2_ was dispersed in 10 mL of Tris–HCl (10 mmol L^−1^) in water through ultrasonic treatment for 10 min until the solution was clear. Then, 1 mL of 1 mg mL^−1^ of DA-HCl was added and stirred for 30 min. Subsequently, 2 MnO_4_ was added to the solution, and the reactants were stirred for 4 h and cenmL of centrifuged. KMnO_4_ was used as raw material, dopamine was used as reducing agent, and MnO_2_ was coated on MoS_2_ surface by REDOX method.

The black powder obtained was washed with deionized water three times to obtain MoS_2_@MnO_2_ [29].

For the preparation of MoS_2_@MnO_2_-PEG, 5 mg of MoS_2_@MnO_2_ samples were dispersed in 10 mL of deionized water, and 2 mL of mPEG-NH_2_ (10 mg mL^−1^) was added. The solution was stirred for 24 h at room temperature, and black powder nanoparticles MoS_2_@MnO_2_-PEG were obtained.

### Characterization

The morphology and structure of the materials were analyzed through transmission electron microscopy (TEM) and scanning electron microscopy (SEM). The crystal structure of the materials was determined using X-ray diffraction (XRD). The synthesized materials were further characterized using X-ray photoelectron spectroscopy (XPS), UV–vis spectroscopy, and Fourier transform infrared (FTIR) spectroscopy.

### Photothermal performance

The photothermal performance of MoS_2_@MnO_2_-PEG was evaluated based on drug concentration, power density, and photostability. To investigate its photothermal properties, the following three aspects were explored: (a) A series of concentration gradients of MoS_2_@MnO_2_-PEG were prepared and irradiated with an 808 nm laser (1.5 W cm^−2^). Temperature changes were monitored every 30 s for 10 min, and the temperature at 0, 1, 3, 5, 7, and 10 min were photographed and recorded with a thermal imager. Then, the subsequent processing was carried out. (b) MoS_2_@MnO_2_-PEG with a concentration of 200 μg mL^−1^ was exposed to lasers with power densities of 1 W cm^−2^, 1.5 W cm^−2^, and 2 W cm^−2^ at 808 nm. Temperature changes were monitored every 30 s for 10 min, and the data were recorded. (c) 0.5 mL of MoS_2_@MnO_2_-PEG (200 μg mL^−1^) was irradiated with a laser switch repeatedly for five cycles (1.5 W cm^−2^), and the temperature was recorded.

### Chemodynamic performance

To detect the formation of ROS, methylene blue (MB) was used as an indicator. (a) The effect of GSH concentration on ROS was explored by dispersing the prepared MoS_2_@MnO_2_-PEG in acetic acid buffer at pH 5.0, adding a series of concentration gradients of GSH (0 mmol L^−1^, 0.5 mmol L^−1^, 1.0 mmol L^−1^), and incubating at 37 °C for 30 min. MB (10 μg mL^−1^) and H_2_O_2_ (100 mmol L^−1^) were added to the solution, and the mixture was incubated at 37 °C for 30 min. The solution was then centrifuged, and the supernatant was taken for UV–Vis absorbance. (b) Two control experiments were conducted to explore the effect of temperature on ROS: one set up at 37 °C and the other at 60 °C. The remaining experiments were performed as described above. (c)To determine the type of ROS produced, the prepared MoS_2_@MnO_2_-PEG was dispersed in acetic acid buffer at pH 5.0, and GSH (1.0 mmol L^−1^) was added. The mixture was incubated at 60 °C for 30 min. Phosphate buffer saline (PBS), MoS_2_@MnO_2_-PEG, Isopropyl alcohol (IPA), NaN_3_, and p-benzoquinone (PBQ) were added successively to capture ·OH, ^1^O_2_, and ·O_2_^−^. IPA captured ·OH, NaN_3_ captured ^1^O_2_, PBQ captured ·O_2_^−^. Finally, MB (10 μg mL^−1^) and H_2_O_2_ (100 mmol L^−1^) were added to the solution, and the mixture was incubated at 60 °C for 30 min.

### Cell experiments

#### Cell uptake

HeLa cells were seeded into a cell culture dish at a density of 1 × 10^5^ and cultured for 24 h. To evaluate the potential of MoS_2_@MnO_2_-PEG nanoparticles for cell fluorescence imaging, RhB was loaded into the nanoparticles. Next, 1.0 mL of the MoS_2_@MnO_2_-PEG nanoparticles dispersed in DMEM medium was added to each confocal dish and incubated for different time periods (0 h, 1 h, 2 h, 4 h, and 6 h). After incubation, the medium was discarded and the cells were washed twice with PBS. DAPI was then added to stain the nucleus, and the cells were incubated for 30 min before observation and photography under a confocal microscope. This protocol allowed us to assess the ability of the MoS_2_@MnO_2_-PEG nanoparticles to label cells and provide clear images of the nucleus for cell fluorescence imaging.

#### Detect ROS generation

HeLa cells were seeded in 6-well plates at a density of 1 × 10^5^ and cultured overnight. After 24 h, the cells were treated with DCFH-DA (1 × 10^−6^ M) for 40 min. To evaluate ROS scavenging ability of MoS_2_@MnO_2_-PEG nanoparticles, three capture agents were used: PBQ (60 mM) as an ·O_2_^−^ trapping agent, IPA (60 mM) as a capture agent for ·OH, and NaN_3_ (60 mM) as a capture agent for ^1^O_2_. Six groups of experiments were set up: (a) PBS group, (b) MoS_2_@MnO_2_-PEG group, (c) MoS_2_@MnO_2_-PEG + Laser group, (d) MoS_2_@MnO_2_-PEG + Laser + IPA group, (e) MoS_2_@MnO_2_-PEG + Laser + PBQ group, and (f) MoS_2_@MnO_2_-PEG + Laser + NaN_3_ group. The fluorescence intensity of DCF was measured to evaluate the ROS scavenging ability of MoS_2_@MnO_2_-PEG nanoparticles. This experimental design allowed us to investigate the effectiveness of MoS_2_@MnO_2_-PEG nanoparticles in scavenging ROS, which has important implications for their potential biomedical applications.

#### In vitro cytotoxicity

To evaluate the cytotoxicity of MoS_2_@MnO_2_-PEG nanoparticles, MTT assay was performed. Different conditions of MoS_2_@MnO_2_-PEG dispersed in DMEM were incubated for 12 h and 24 h. MTT (0.5 mg mL^−1^) was added to each well and incubated for 4 h; the medium was discarded, followed by the addition of 150 μL DMSO. The absorbance at 490 nm was measured to determine cell viability.

To assess the type of ROS that trigger apoptosis, the Calcein-AM/PI method was used. HeLa cells were seeded at a density of 1 × 10^5^ in 6-well plates and incubated for 24 h. Five sets of experiments were conducted: (a) PBS group, (b) MoS_2_@MnO_2_-PEG group, (c) PBQ (60 mM) + MoS_2_@MnO_2_-PEG group, (d) IPA (60 mM) + MoS_2_@MnO_2_-PEG group, and (e) NaN_3_ (60 mM) + MoS_2_@MnO_2_-PEG group. After 4 h of incubation, DMEM was discarded and Calcein-AM/PI probes were added, followed by incubation in the dark for 30 min. The results were recorded using fluorescence microscopy. This experimental design allowed us to investigate the cytotoxicity of MoS_2_@MnO_2_-PEG nanoparticles and the type of reactive oxygen species that trigger apoptosis, which are essential for their potential biomedical applications.

#### Tumor model

Female Balb/c mice (ages: about 6 weeks; body weights: about 18 g) were selected as animal models to mimic HeLa cancer cells. The mice were purchased from Beijing Vital River Laboratory Animal Technology Co., Ltd. and approved by the Animal Ethics and Use Committee of Shanxi Medical University (IACUC 2017–018). The study was conducted in compliance with ethical guidelines and the mice were euthanized at the end of the treatment.

#### In vivo biocompatibility assay

Female Balb/c mice were divided into four groups: (a) PBS; (b) PBS + Laser; (c) MoS_2_@MnO_2_-PEG, and (d) MoS_2_@MnO_2_-PEG + Laser. The mice received an intravenous injection of the drug (5 mg kg^−1^, 200 μL) and their body weights were measured every two days. After 14 days of treatment, the main organs of the mice were harvested and stained with hematoxylin and eosin (H&E) to assess any potential histological changes. This experimental design allowed us to investigate the potential toxicity of MoS_2_@MnO_2_-PEG nanoparticles and their safety for use in biomedical applications.

### Imaging experiments

#### In vitro MR imaging

To investigate the relationship between the concentration of MoS_2_@MnO_2_-PEG and T1-weighted MRI signal under in vitro conditions, a series of concentration gradients were prepared for excess GSH-pretreated MoS_2_@MnO_2_-PEG in 200 μL centrifuge tubes. The MRI signals of the samples were then detected to assess the linear relationship between the concentration of MoS_2_@MnO_2_-PEG and T1-weighted MRI signal. This experimental design allowed us to evaluate the potential of MoS_2_@MnO_2_-PEG nanoparticles as contrast agents for MRI imaging.

#### In vivo MR imaging

To evaluate the potential of MoS_2_@MnO_2_-PEG nanoparticles as contrast agents for T_1_-weighted MR imaging in vivo, the nanoparticles were injected into tumor-bearing nude mice through the tumor. T_1_-weighted MR imaging was then performed, and the gray values of the MR signal were recorded. This experimental design allowed us to assess the ability of MoS_2_@MnO_2_-PEG nanoparticles to enhance the contrast of MR imaging in vivo, which is important for their potential biomedical applications.

## Results and discussion

### Characterization

**Fig. 1:**
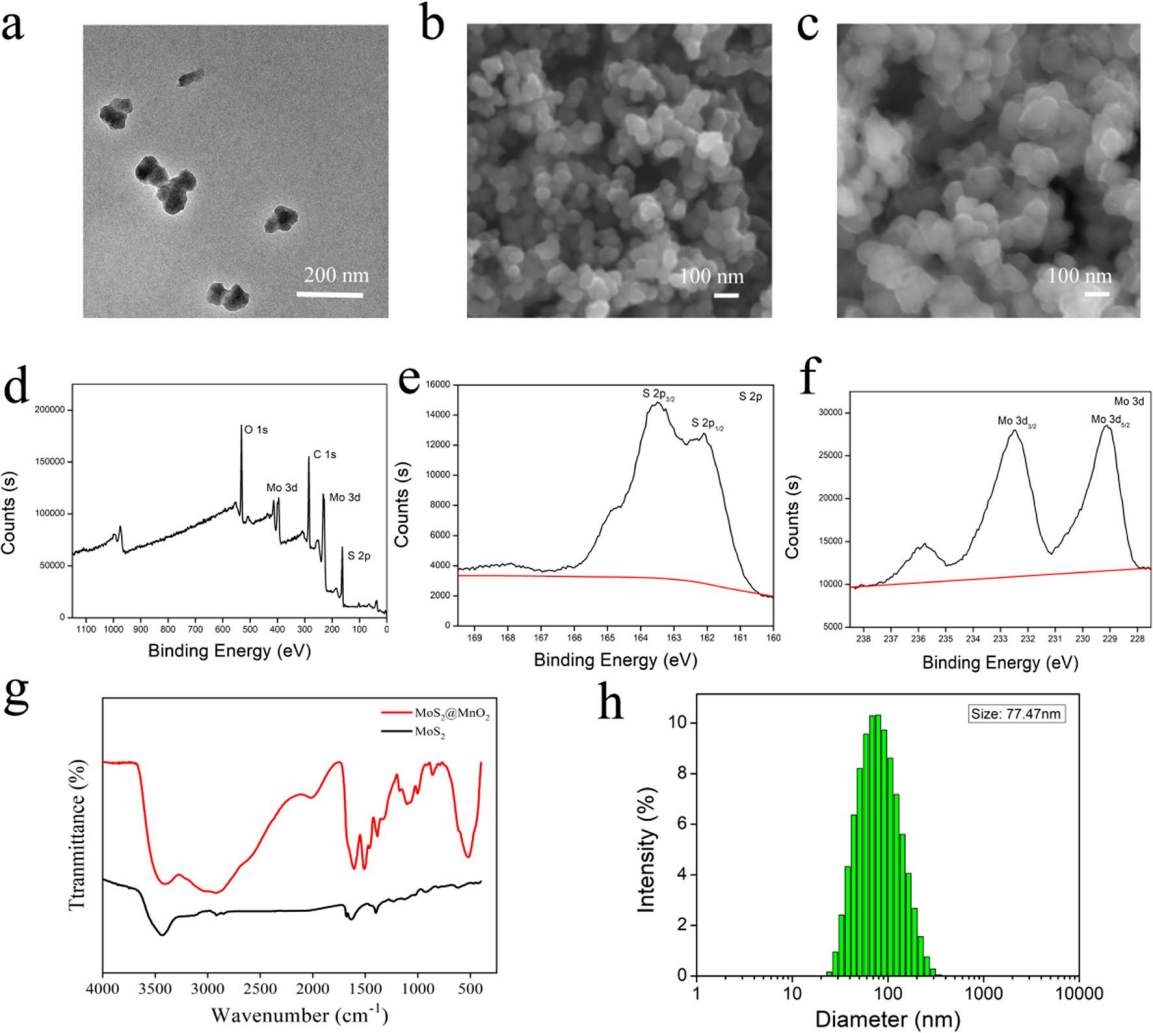
Characterization of MoS_2_@MnO_2_. **a** TEM images of MoS_2_@MnO_2_. **b** SEM images MoS_2_, and **c** MoS_2_@MnO_2_. **d** XPS spectra of MoS_2_, **e** XPS spectra of S 2p, **f** Mo 3d. **g** FTIR spectrum of MoS_2_@MnO_2_. **h** The hydrated particle size of MoS_2_@MnO_2_

The morphology of the nanoparticles was characterized by TEM, SEM, and DLS. The TEM results (Fig. 1a) demonstrated that the synthesized MoS_2_@MnO_2_ was a homogeneous spherical particle with a size of approximately 80 nm. The hydrated particle size (Fig. 1h) of the nanoparticles was measured to be 77.47 nm, consistent with the TEM results. The SEM images of MoS_2_ (Fig. 1b) and MoS_2_@MnO_2_ (Fig. 1c) showed the in-situ growth of MnO_2_ on the surface of MoS_2_, confirming the successful preparation of MoS_2_@MnO_2_. The potential (Fig. S1) of MoS_2_, MoS_2_@MnO_2_, and MoS_2_@MnO_2_-PEG was measured. The surface potential of MoS_2_ was − 40 mV, while the surface potential of MoS_2_@MnO_2_-PEG, formed after wrapping MnO_2_ on its surface by redox reaction, was − 23.2 mV. To improve its biocompatibility, the surface potential of MoS_2_@MnO_2_-PEG was modified to − 11.4 mV by electrostatic modification of mPEG-NH_2_ on its surface. The potential test results confirmed the successful preparation of MoS_2_@MnO_2_-PEG.

The successful synthesis of MoS_2_ was confirmed by XPS (Fig. 1d), which revealed peaks for Mo, S, C, and O. The two main peaks of Mo 3d_5/2_ and Mo 3d_3/2_ of MoS_2_ were observed at 231.67 and 228.45 eV (Fig. 1f), respectively. The high-resolution spectrum of S 2p showed the binding energy of S at 2p_3/2_ and S 2p_1/2_ at 162.6 eV and 161.8 eV, respectively (Fig. 1e). These results confirmed the successful preparation of MoS_2_.

The successful preparation of MoS_2_ and MoS_2_@MnO_2_ was further characterized by infrared spectroscopy (Fig. 1g), which showed that the peak of MoS_2_ was located at about 467 cm^−1^, corresponding to the characteristic peak of Mo–S vibration, confirming the successful preparation of MoS_2_. Distinct peaks of Mn–O vibrations at 550 cm^−1^ were observed in the infrared spectrum of MoS_2_@MnO_2_, characterizing the successful preparation of MoS_2_@MnO_2_.

**Fig. 2:**
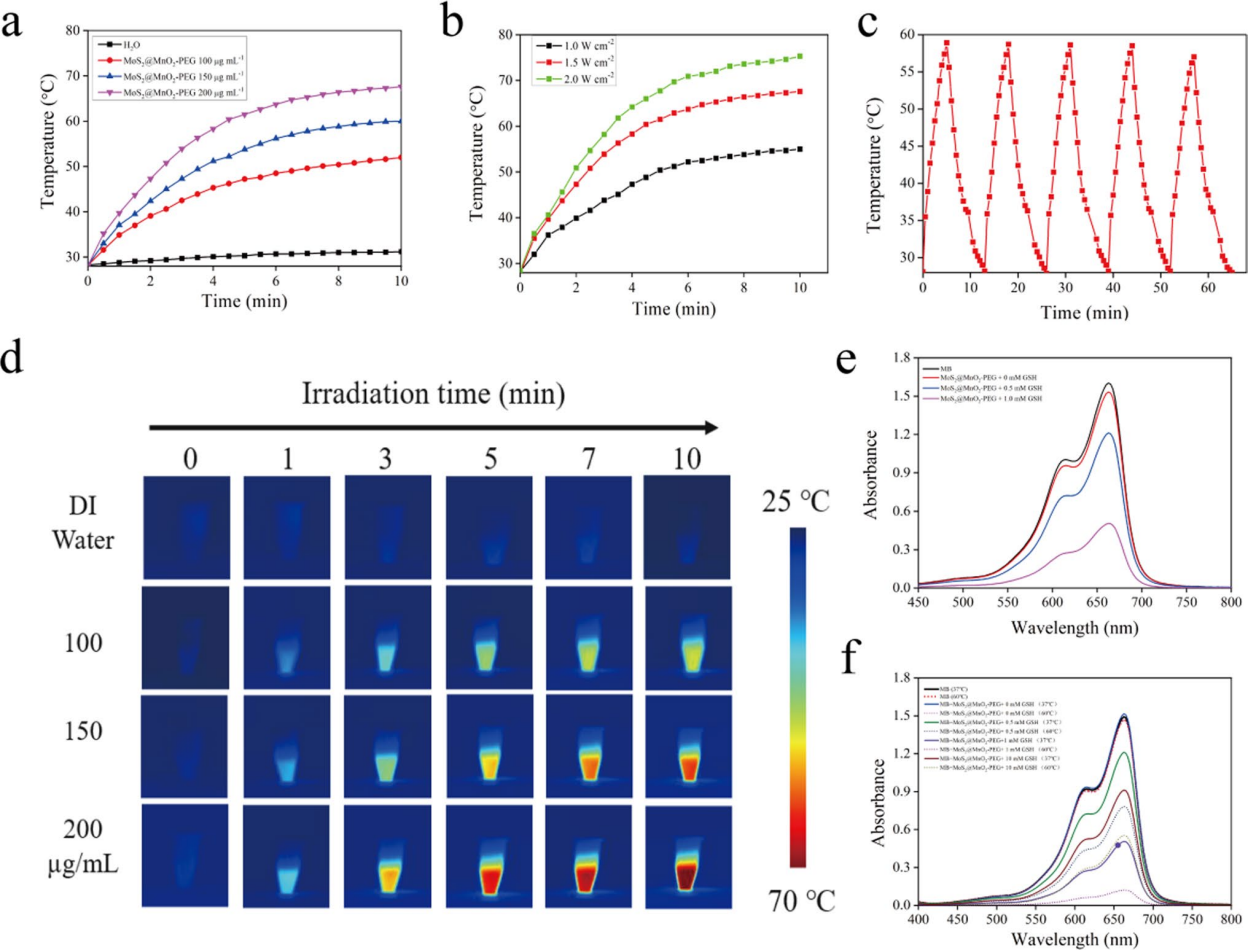
**a** Temperature change curves of different concentrations of MoS_2_@MnO_2_-PEG under 808 nm laser irradiation. **b** Temperature change curves of MoS_2_@MnO_2_-PEG dispersion (200 μg mL^−1^) at different laser power densities. **c** temperature plot of MDMP dispersion irradiated by an 808 nm laser for five on–off cycles. **d** Infrared thermal imaging pictures of different concentrations of MoS_2_@MnO_2_-PEG at different irradiation times. **e** Investigate the effect of GSH concentration on ROS content produced by MoS_2_@MnO_2_-PEG. **f** The influence of temperature on the ROS content

The UV–Vis absorption spectroscopy (Figure S2) of MoS_2_@MnO_2_-PEG revealed that it was widely absorbed in the near-infrared I region, and the absorbance increased with drug concentration, demonstrating its potential as a photothermal agent. The photothermal properties of MoS_2_@MnO_2_-PEG were investigated by exploring the influence of concentration (Fig. 2a) and laser power (Fig. 2b) on the temperature changes of MoS_2_@MnO_2_-PEG under 808 nm laser irradiation. The temperature increased with the concentration of MoS_2_@MnO_2_-PEG, and at 200 μg mL^−1^, the temperature increased from 27 to 65 °C after 10 min of 1.5 W cm^−2^ laser irradiation, achieving the purpose of photothermal treatment of tumors. Therefore, 200 μg mL^−1^ MoS_2_@MnO_2_-PEG was used for subsequent experiments. The temperature changes of MoS_2_@MnO_2_-PEG were positively correlated with the laser power (1 W cm^−2^, 1.5 W cm^−2^, and 2 W cm^−2^), and after 1.5 W cm^−2^ laser irradiation, the temperature of the solution increased from 27 to 65 °C, achieving the purpose of PTT treatment of tumors. Thus, a laser with 1.5 W cm^−2^ power was used as the experimental power in subsequent experiments.

The NIR thermal imager was used to record the temperature of different concentrations of MoS_2_@MnO_2_-PEG (0 μg mL^−1^, 100 μg mL^−1^, 150 μg mL^−1^, and 200 μg mL^−1^) at different times (0 min, 1 min, 3 min, 5 min, 7 min, 10 min) under 1.5 W cm^−2^ 808 nm laser irradiation (Fig. 2d). The photothermal properties of the material gradually increased with the increase in material concentration, and the photothermal properties increased with the laser irradiation time.

The study investigated the photostability and photothermal stability of MoS_2_@MnO_2_-PEG. The results showed that the absorbance of MoS_2_@MnO_2_-PEG did not change significantly before and after laser irradiation (Figure S3), indicating excellent photostability. Moreover, the temperature response curve of MoS_2_@MnO_2_-PEG remained unchanged after five cycles of laser heating and cooling (Fig. 2c), demonstrating its exceptional photothermal cycle stability. Moreover, according to Roper’s method, the photothermal conversion efficiency (η) of MoS_2_@MnO_2_-PEG (200 μg mL^−1^) was calculated to be around 27.1% (Figure S4), demonstrating its excellent light-to-heat conversion capability [30]. These findings suggested that the synthesized MoS_2_@MnO_2_-PEG nanocomposite possesses superior photostability and photothermal stability properties that are relevant for various biomedical and industrial applications.

### Chemodynamic performance

In this study, we evaluated the chemodynamic performance of MoS_2_@MnO_2_-PEG nanoparticles in the tumor microenvironment (TME). The MnO_2_ coating on the outer layer of the nanoparticles facilitated the reaction with excess GSH in the TME to produce Mn^2+^. Further, Mn^2+^ decomposed H_2_O_2_, ultimately generating cytotoxic ROS34. Using MB as a ROS probe, we investigated the effect of GSH concentration on ROS content produced by MoS_2_@MnO_2_-PEG (Fig. 2e). Gradual decrease in absorbance of MB at 660 nm with increasing GSH concentration indicated a corresponding increase in ROS content generated by the MoS_2_@MnO_2_-PEG system, thus verifying its chemodynamic performance. In order to confirm the synergistic enhancement phenomenon of photothermal therapy and chemodynamic therapy,35,36 we explored the influence of temperature on the ROS content (Fig. 2f), which showed that the absorbance value of MB at 660 nm was significantly lower at 60 °C than at 37 °C, indicating that the system produced more active oxygen species.

To investigate the types of ROS produced by the nanoparticles, we used PBQ as a trapping agent for O^2−^, IPA as a capture agent for ·OH, and NaN_3_ as a trapping agent for ^1^O_2_. Our findings suggested that MoS_2_@MnO_2_-PEG nanoparticles primarily produce ·OH instead of O^2−^,^1^O_2_ (Figure S5), as shown by the increased absorbance value of MB at 660 nm in the MoS_2_@MnO_2_-PEG + IPA treatment group but no significant difference in the absorbance value of MB at 660 nm in the MoS_2_@MnO_2_-PEG + NaN_3_ group compared to the MoS_2_@MnO_2_-PEG + PBQ group. These findings highlighted the potential of MoS_2_@MnO_2_-PEG nanoparticles as an effective chemodynamic therapy agent for cancer treatment.

### Cellular experiments

#### Cell uptake

**Fig. 3:**
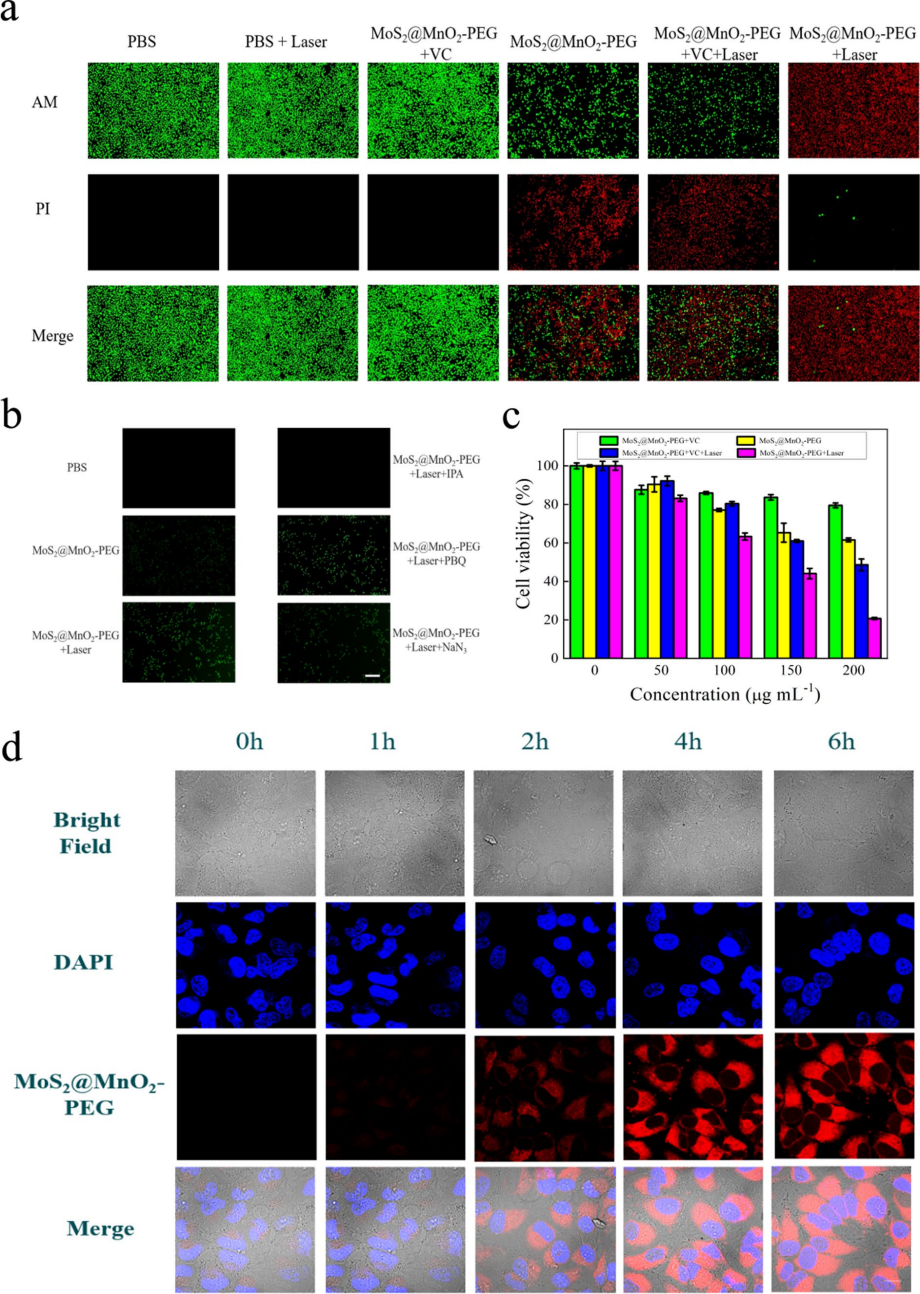
**a** Calcein-AM/PI dual staining for identifying the live or dead cells. **b** The ability of MoS_2_@MnO_2_-PEG to generate ROS at the cellular level. **c** HeLa cells viability after incubation with different groups. **d** Cellular uptake of MoS_2_@MnO_2_-PEG observed by CLSM for different periods

To investigate the cellular uptake of MoS_2_@MnO_2_-PEG nanoparticles and evaluate their ability to generate ROS at the cellular level, we conducted a series of experiments. First, we used rhodamine B to label the surface of MoS_2_@MnO_2_-PEG nanoparticles and observed their uptake by HeLa cells using laser confocal microscopy (Fig. 3d). The intracellular fluorescence increased with the extension of the incubation time, indicating that the uptake of MoS_2_@MnO_2_-PEG by cells gradually increased. 3.4.2.ROS generation.

#### ROS generation

Next, we evaluated the ability of MoS_2_@MnO_2_-PEG to generate ROS at the cellular level using the DCFH-DA probe (Fig. 3b). Compared with the PBS group, fluorescence was detectable in the cells of the MoS_2_@MnO_2_-PEG group, indicating that ROS was generated in the system. Moreover, the fluorescence intensity of the system after laser irradiation was stronger in the MoS_2_@MnO_2_-PEG + Laser group than in the MoS_2_@MnO_2_-PEG group, indicating that the system had a PTT-enhanced CDT effect. The types of ROS produced by MoS_2_@MnO_2_-PEG were also examined using different ROS trap detection systems. The fluorescence of the MoS_2_@MnO_2_-PEG + PBQ group and the MoS_2_@MnO_2_-PEG + NaN_3_ group was strong, while the fluorescence of the MoS_2_@MnO_2_-PEG + IPA group almost disappeared, indicating that the type of reactive oxygen species produced by MoS_2_@MnO_2_-PEG was ·OH.

#### In vitro cytotoxicity

We then assessed the cytotoxicity of MoS_2_@MnO_2_-PEG using normal human cells (HcerEpic Cell) and HeLa cells (Fig. S6). MoS_2_@MnO_2_-PEG at 400 μg mL^−1^ was incubated with normal human cells for 12 h or 24 h, and the survival rate of cells was more than 80%, indicating that MoS_2_@MnO_2_-PEG had good biological safety and was non-toxic to normal cells. However, the survival rate of 200 μg mL^−1^ MoS_2_@MnO_2_-PEG-treated HeLa cells was reduced to 60% after only 12 h incubation due to the release of Mn^2+^ in response to excessive GSH in the tumor microenvironment.

To evaluate the phototoxicity of MoS_2_@MnO_2_-PEG to tumor cells (Fig. 3c), we conducted experiments using VC + MoS_2_@MnO_2_-PEG-PEG and VC + MoS_2_@MnO_2_-PEG + Laser groups. The survival rate of VC + MoS_2_@MnO_2_-PEG-PEG group cells was above 80%, indicating that the cells were protected from CDT because VC had the effect of clearing ROS. However, the cell survival rate of VC + MoS_2_@MnO_2_-PEG + Laser group was about 50%, indicating that MoS_2_@MnO_2_-PEG had PTT properties. Moreover, the survival rate of cells in the MoS_2_@MnO_2_-PEG + Laser group was about 20%, indicating that PTT/CDT synergistic therapy had a better effect on tumor cell killing.

Finally, we visually assessed the cell-killing properties of MoS_2_@MnO_2_-PEG using Calcein-AM/PI staining. The experimental results demonstrated (Fig. 3a) that the cells of the control groups, PBS and PBS + Laser, exhibited robust viability and the laser illumination alone did not induce cell death. The addition of VC in the VC + MoS_2_@MnO_2_-PEG group conferred protection against CDT through effective scavenging of ROS. Notably, the presence of MoS_2_@MnO_2_-PEG induced partial cell death, indicating the effectiveness of CDT. Furthermore, the introduction of laser irradiation to the VC + MoS_2_@MnO_2_-PEG group confirmed the potent PTT capabilities of MoS_2_@MnO_2_-PEG, with pronounced cell death observed. Most notably, the PTT/CDT combination therapy had a significantly enhanced tumor-killing effect, as evidenced by considerable cell death observed in the VC + MoS_2_@MnO_2_-PEG + Laser group. Additionally, the type of free radicals generated was identified as primarily ·OH using a trapping agent (Fig. S7).

### Imaging experiments

#### MR imaging

**Fig. 4:**
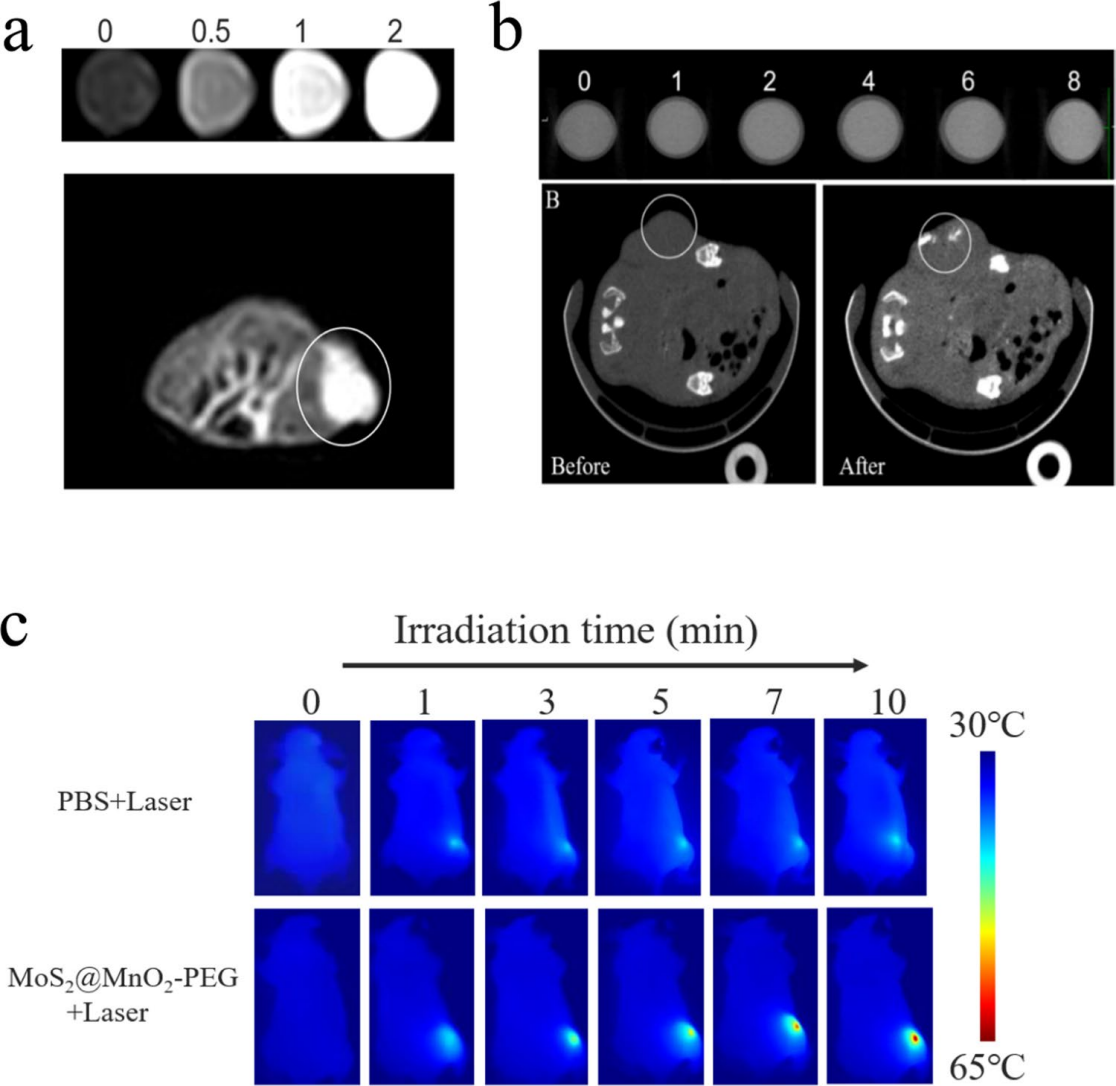
**a** T1-weighted MR images with multiple concentrations and intratumoral T_1_-weighted MR images of MoS_2_@MnO_2_-PEG. **b** CT images with various concentrations and intratumoral CT images of MoS_2_@MnO_2_-PEG. **c** Thermal imaging images of different groups

Previous studies have demonstrated that the Mn element possesses T1 imaging capabilities, which have been effectively imparted to MoS_2_@MnO_2_-PEG for MRI applications. In order to evaluate the potential of MoS_2_@MnO_2_-PEG as an NMR agent, in vitro nuclear magnetic imaging was conducted, with varying concentrations of nanoparticles subjected to pretreatment with excess GSH and incubation for 0.5 h. The resulting T1 NMR signal was observed to increase in accordance with material concentration (Fig. 4a). Subsequently, MoS_2_@MnO_2_-PEG was administered via tumor injection in tumor-bearing nude mice, and in vivo T1 imaging was performed. The resulting T1 signal at the tumor site was significantly different from other sites, indicating the excellent T1 MRI ability of MoS_2_@MnO_2_-PEG.

#### CT imaging

The high atomic number of Mo and its ability to decay X-rays make it an effective CT imaging agent. This study aimed to evaluate the potential of MoS_2_@MnO_2_-PEG as a CT contrast agent through in vitro and in vivo CT imaging. A range of MoS_2_@MnO_2_-PEG concentrations were prepared to assess its in vitro CT imaging effect, and it was observed that the brightness increased in accordance with increasing MoS_2_@MnO_2_-PEG concentration, confirming its good CT imaging ability. In comparison with the tumor site prior to the injection of composite materials, the CT signal at the tumor site after MoS_2_@MnO_2_-PEG injection was enhanced, indicating its good in vivo CT imaging ability (Fig. 4b).

#### Photothermal imaging

The photothermal imaging capabilities of MoS_2_@MnO_2_-PEG were investigated in vivo by injecting it into tumor-bearing nude mice, followed by irradiation of the tumor site with an 808 nm laser (1.5 W cm^−2^) for 10 min (Fig. 4c). The temperature of the tumor site in the experimental group increased by 25 °C, while little change was observed in the control group, indicating the excellent photothermal imaging ability of MoS_2_@MnO_2_-PEG.

### Antitumor effect in vivo

**Fig. 5:**
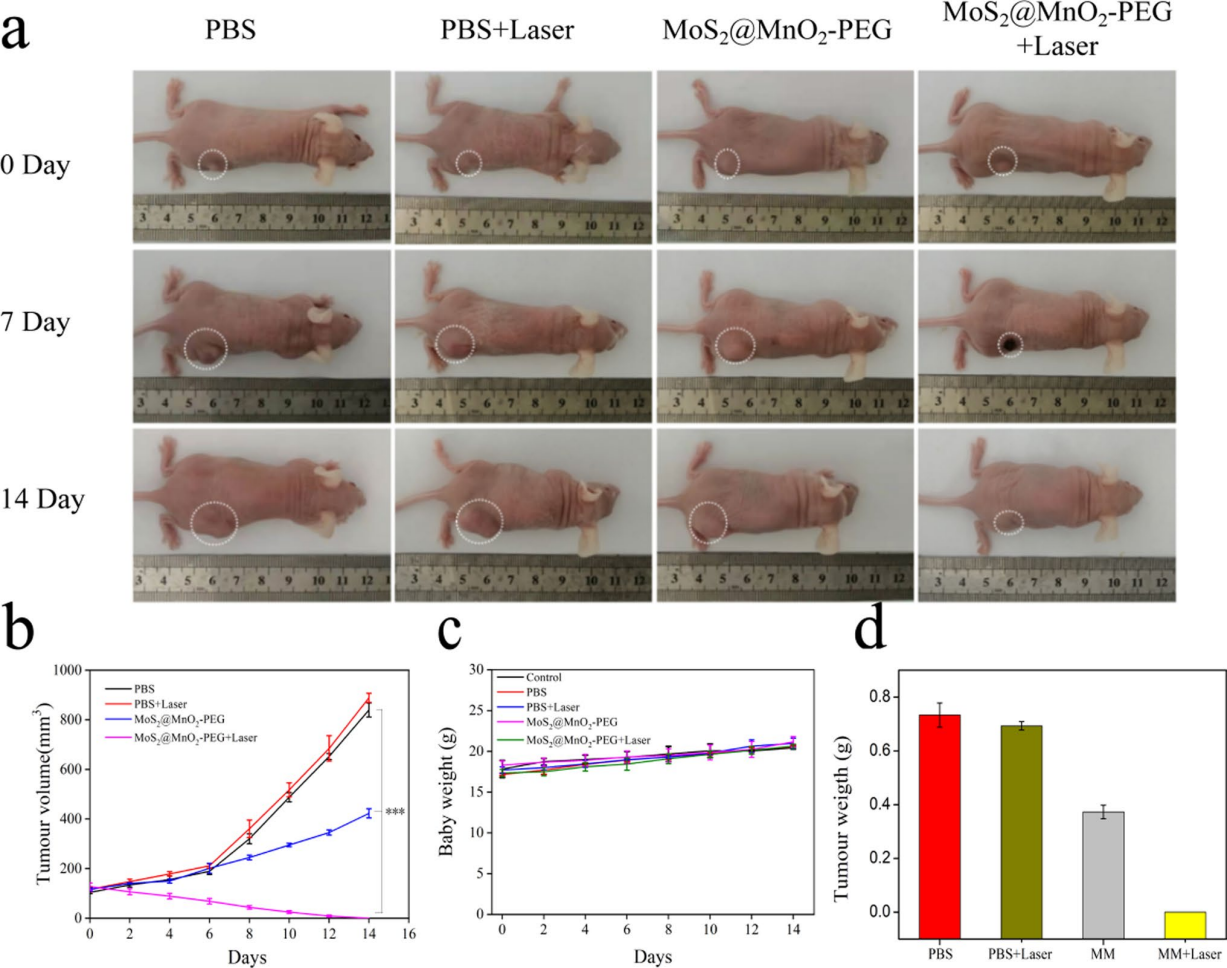
**a** Tumor changes during 14 days of PBS, PBS + Laser, MoS_2_@MnO_2_-PEG, and MoS_2_@MnO_2_-PEG + Laser treatment. **b** tumor volume, **c** body weight, and **d** tumor weight after the treatment of PBS, PBS + Laser, MoS_2_@MnO_2_-PEG, and MoS_2_@MnO_2_-PEG + Laser

In order to assess the antitumor effect in vivo, HeLa cells were inoculated to establish a nude mouse model, with the tumor volume increasing to approximately 100 mm^3^. The mice were divided into four groups: PBS group, PBS + Laser group, MoS_2_@MnO_2_-PEG group, and MoS_2_@MnO_2_-PEG + Laser group, with intratumoral injection and laser irradiation (1.5 W cm^−2^) at 808 nm for 10 min administered in the latter two groups (Fig. 5a, b, d). During the 14-day treatment period, the tumors in the MoS_2_@MnO_2_-PEG group exhibited a smaller size due to the CDT effect, measuring approximately 400 mm^3^. The tumors in the MoS_2_@MnO_2_-PEG plus laser group were eliminated.

**Fig. 6:**
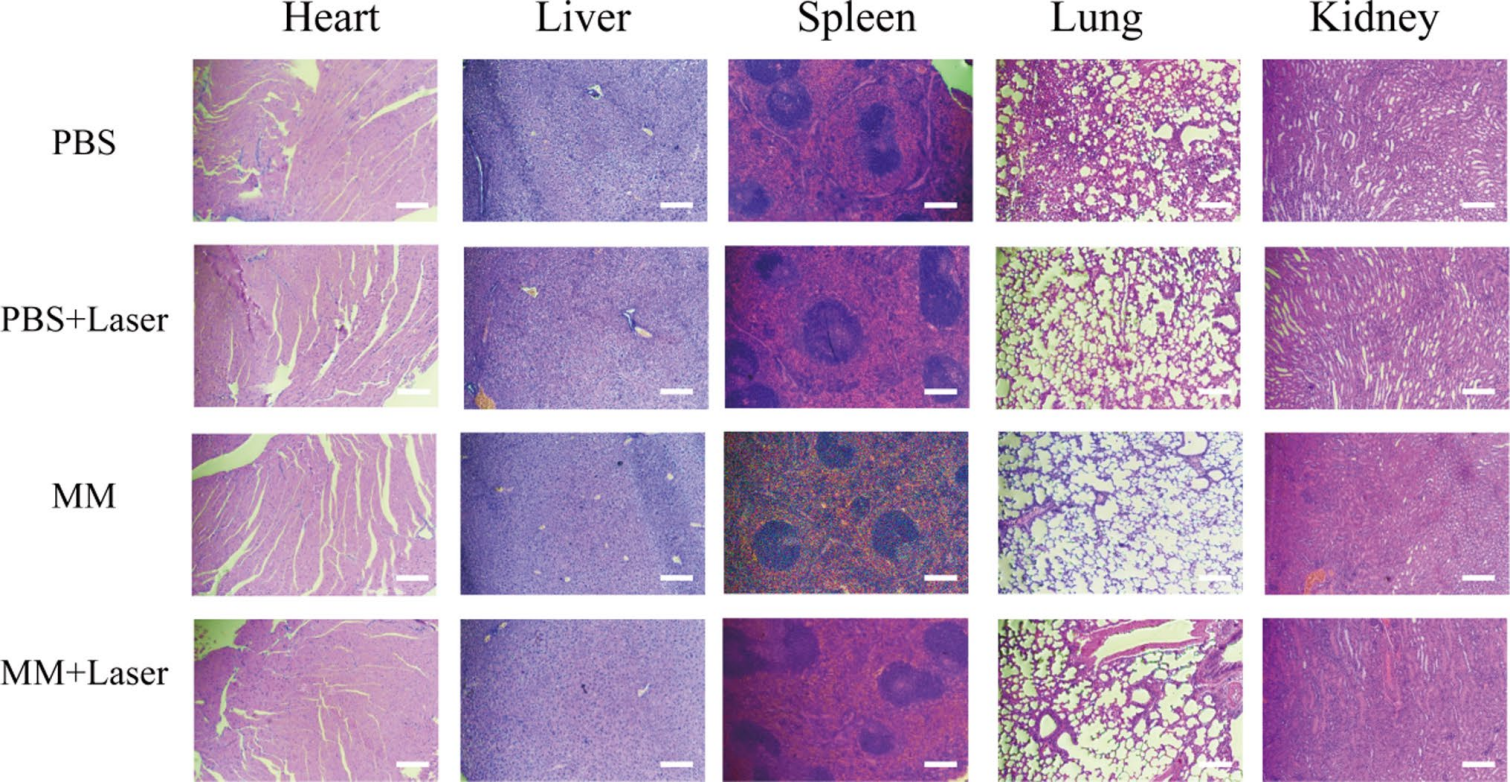
H&E staining images of main organs (heart, liver, spleen, lungs, and kidneys) of HeLa tumor-bearing mice in the groups of PBS, PBS + Laser, MoS_2_@MnO_2_-PEG, and MoS_2_@MnO_2_-PEG + Laser

Following treatment, the nude mice were euthanized, and their main organs and tumor tissues were collected for analysis. The weight of the mice in all four groups increased slightly during the treatment period (Fig. 5c), and no deaths occurred, indicating the good biocompatibility of MoS_2_@MnO_2_-PEG. Liver and renal function in the experimental group showed no significant difference compared to the control group (Fig. S8). Pathological sections were analyzed to assess the effects of MoS_2_@MnO_2_-PEG on the heart, liver, spleen, lungs, and kidneys of nude mice, and no apparent abnormalities were observed in any of the four groups (Fig. 6), indicating the excellent biological safety of MoS_2_@MnO_2_-PEG.

## Conclusion

This study presented a diagnostic and therapeutic integrated nanosystem, MoS_2_@MnO_2_-PEG, which demonstrated excellent performance in PTI, MRI and CT multimodal imaging, and PTT/CDT synergistic therapeutic effects. In vitro tests confirmed the successful preparation of the material and its favorable PTT and CDT properties. MoS_2_@MnO_2_-PEG exhibited high cellular uptake rates, lower toxicity to normal cells, and strong tumor cell-killing ability in cell uptake experiments, demonstrating its excellent antitumor performance. Furthermore, our results from PTI, MRI, and CT imaging confirmed the ability of MoS_2_@MnO_2_-PEG for multimodal imaging. Animal experiments established the biological safety and excellent therapeutic effects of MoS_2_@MnO_2_-PEG on tumor-bearing nude mice. Our approach integrating CDT/PTT synergies through MoS_2_@MnO_2_-PEG held great significance in promoting the integration of tumor diagnosis and treatment and provided a new strategy for tumor treatment.

### Supplementary Information

Supplementary file 1

## Data Availability

Data will be made available on request.

## Code availability

Source code is available upon request from the corresponding author.
