# Supplementary material for: Multimodal imaging and photothermal/chemodynamic therapy of cervical cancer using GSH-responsive MoS2@MnO2 theranostic nanoparticles
Source: Discov Nano. 2023 Sep 29;18(1):122. doi: 10.1186/s11671-023-03902-9 (PMC10541390; doi:10.1186/s11671-023-03902-9)
Supplement: Supplementary file 1 [file 11671_2023_3902_MOESM1_ESM.docx]

**Supporting Information**

**Multimodal imaging and photothermal/chemodynamic therapy of cervical cancer using GSH-responsive MoS_2_@MnO2 theranostic nanoparticles**

Runrun Shao^1^, Xiaofang Qiao^2^, Linlin Cao^2^, Jianliang Man^1^, Lingyun Guo^1^,Lanlan Li^1^,Wen Liu^1^,Lihong Li^1^,Bin Wang^1^, Lixia Guo^1^, Sufang Ma^1^, Boye Zhang^1^, Haojiang Wang^1^, and Lili Yan^1^*

^1^College of Basic Medicine University, Shanxi Medical Univerity, Taiyuan, 030000, P. R. China.

^2^Henan Center For Drug Evaluation and Inspection, Henan, 450000, P. R. China.

**Supplementary Figures**


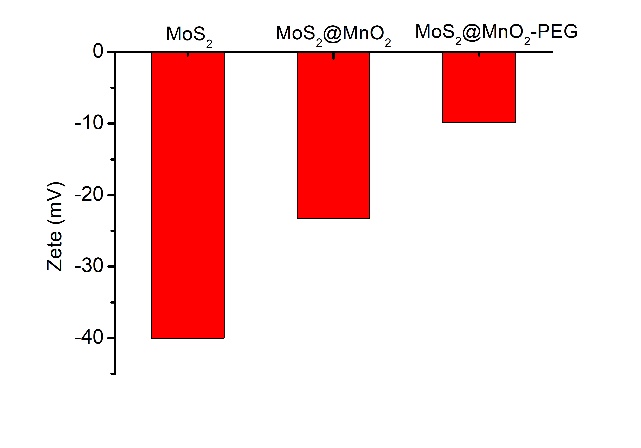


Figure S1.The Zeta potential of MoS_2_, MoS_2_@MnO_2_, and MoS_2_@MnO_2_-PEG.


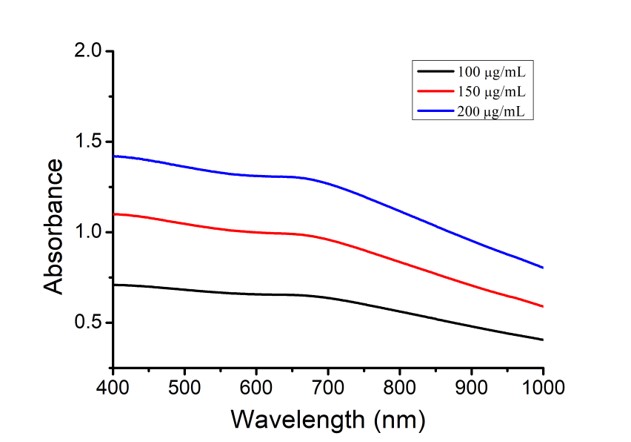


**Figure S2**.UV absorption MoS_2_@MnO_2_ at different concentrations


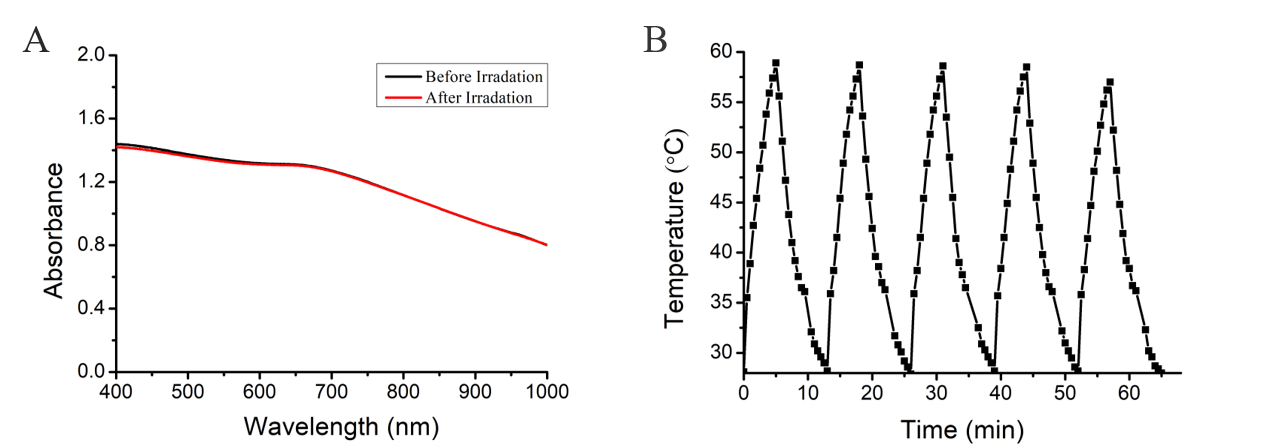


**Figure S3**.The photostability of MoS_2_@MnO_2_.


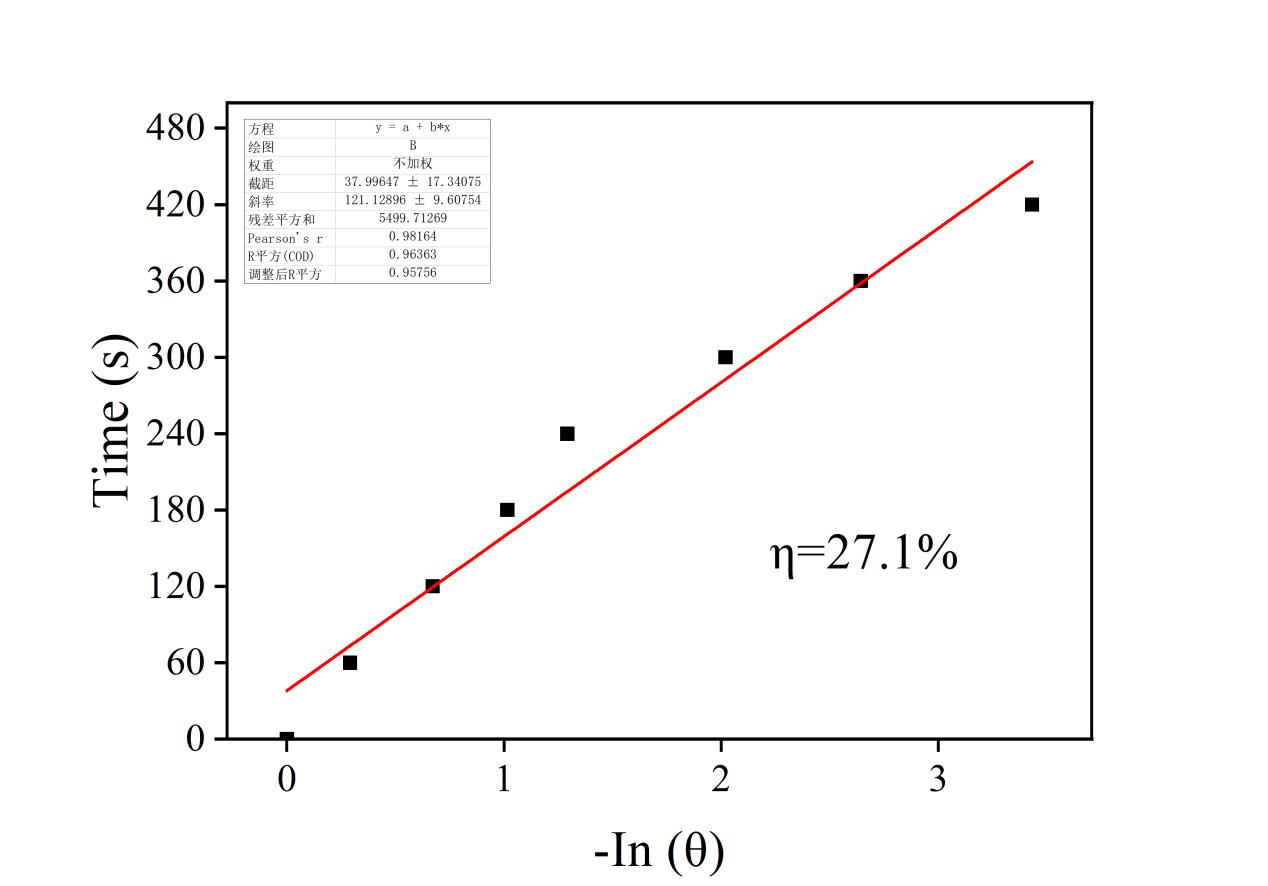


**Figure S4. Plot of the cooling time as a function of the negative natural logarithm of the temperature driving force and** photothermal conversion efficiency (η).


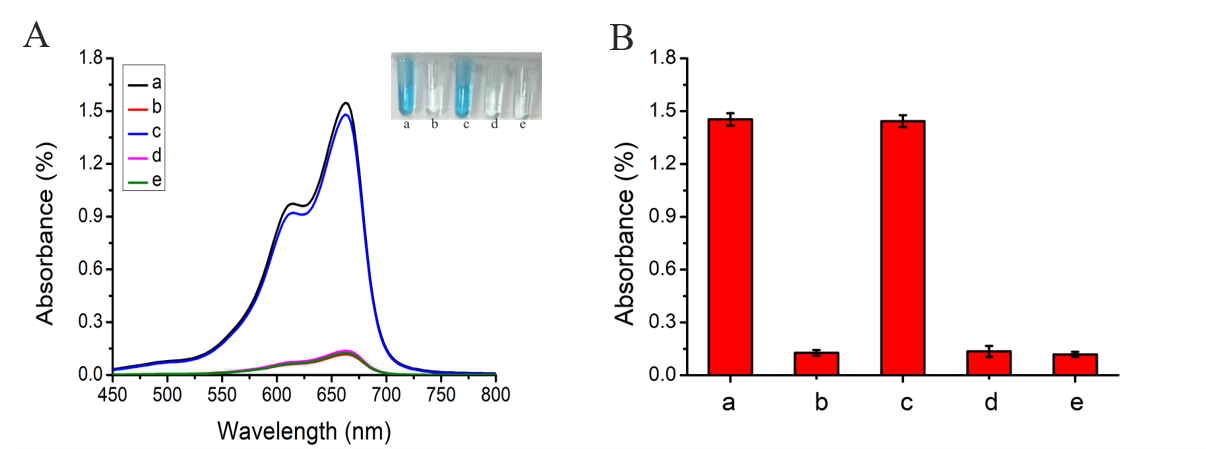


**Figure S5**. (A) Add different traps to detect the type of reactive oxygen species; (B) UV-Vis chart. where (a：MB；b：MB+MoS_2_@MnO_2_-PEG+H_2_O_2_+HCO_3_^-^+1.0 mM GSH；c：MB+MoS_2_@MnO_2_-PEG+H_2_O_2_+HCO_3_^-^+1.0 mM GSH+IPA；d：MB+MoS_2_@MnO_2_-PEG+H_2_O_2_+ HCO_3_^-^+1.0 mM GSH+PBQ；e：MB+MoS_2_@MnO_2_-PEG+H_2_O_2_+HCO_3_^-^+1.0 mM GSH+NaN_3_））。


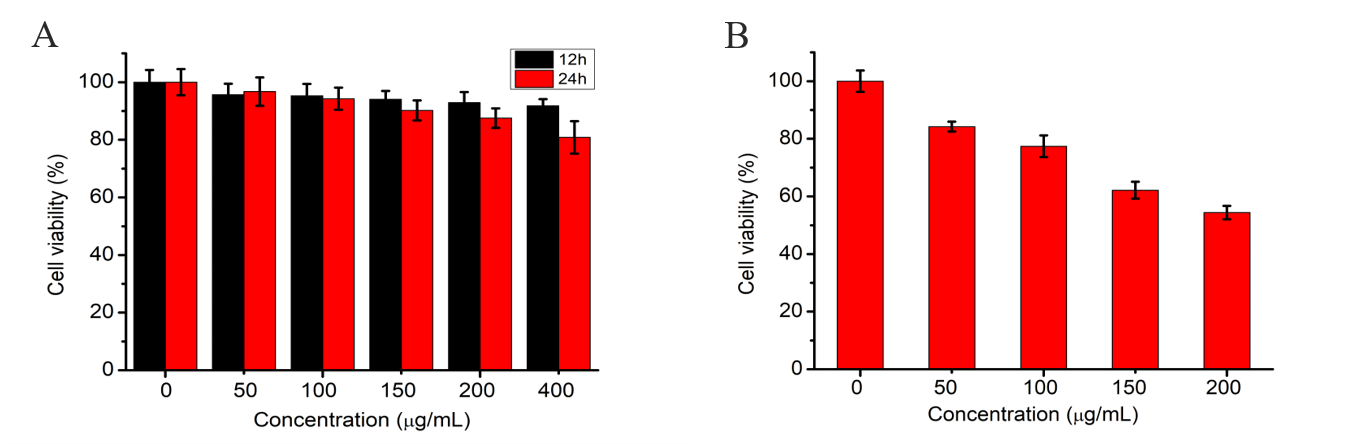


**Figure S6**.(A) Effects of different concentrations of MoS_2_@MnO_2_-PEG on HcerEpic cell viability;

1. Effect of different concentrations of MoS_2_@MnO_2_-PEG on HeLa cell viability.


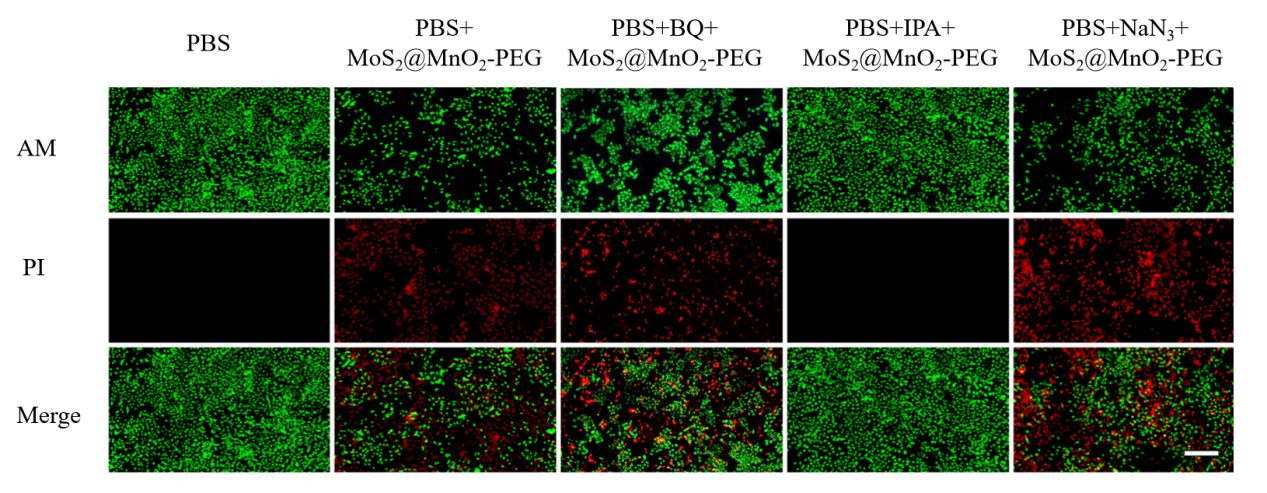


**Figure S7**.Calcein-AM/PI staining observation method to verify the type of reactive oxygen species that trigger apoptosis (scale bar : 100 μm)


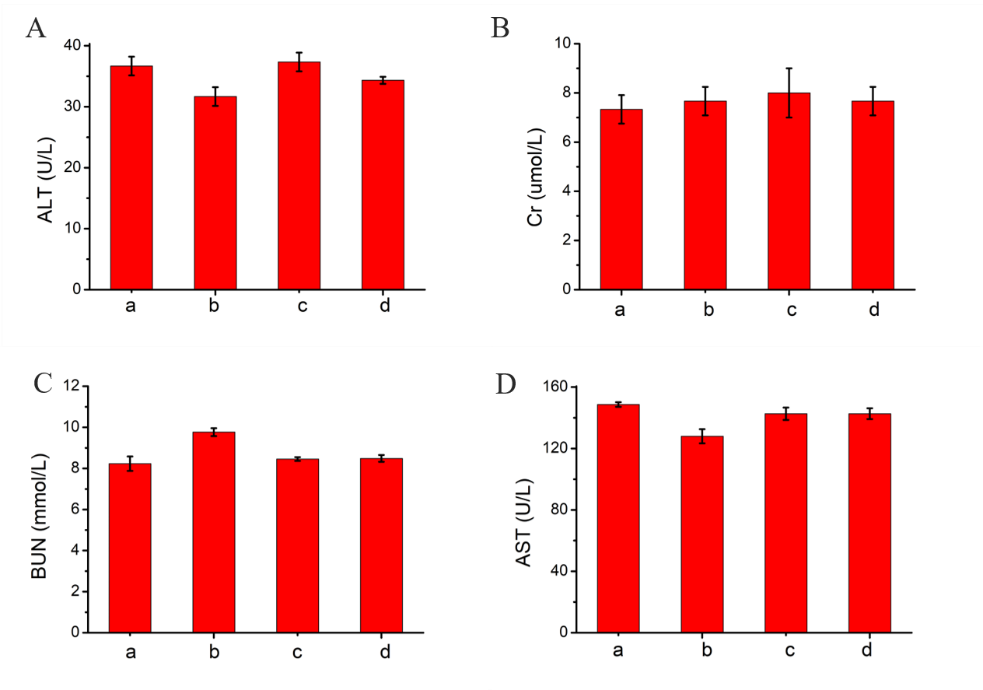


**Figure S8**.Statistical chart of serum biochemical indexes in nude mice. (A) alanine aminotransferase (ALT); (B) creatinine (Cr); (C) urea nitrogen (BUN); (D) Aspartate aminotransferase (AST). (a：PBS；b：PBS+Laser；c：MoS_2_@MnO_2_-PEG；d：MoS_2_@MnO_2_-PEG+Laser)。
